# Evaluating new paralysis, mortality, and readmission among subgroups of patients with spinal epidural abscess: A latent class analysis

**DOI:** 10.1371/journal.pone.0238853

**Published:** 2020-09-11

**Authors:** Patrick C. M. Brown, Gina M. Phillipi, Caroline King, Mary Tanski, Peter Sullivan

**Affiliations:** 1 School of Medicine, Oregon Health & Science University, Portland, OR, United States of America; 2 School of Medicine, Department of Biomedical Engineering, Oregon Health & Science University, Portland, OR, United States of America; 3 Oregon Health & Science University, Portland, OR, United States of America; 4 Department of Emergency Medicine, Oregon Health & Science University, Portland, OR, United States of America; 5 Department of Internal Medicine, Oregon Health & Science University, Portland, OR, United States of America; Texas A&M University, UNITED STATES

## Abstract

**Background:**

Spinal epidural abscess (SEA) is increasing in incidence; this not-to-miss diagnosis can cause significant morbidity and mortality, particularly if diagnoses are delayed. While some risk factors for SEA and subsequent mortality have been identified, the SEA patient population is clinically heterogeneous and sub-populations have not yet been characterized in the literature. The primary objective of this project was to identify characteristics of subgroups of patients with SEA. The secondary objective was to identify associations between subgroups and three clinical outcomes: new onset paralysis, in-hospital mortality, and 180-day readmission.

**Methods:**

Demographics and comorbid diagnoses were collected for patients diagnosed with SEA at an academic health center between 2015 and 2019. Latent class analysis was used to identify clinical subgroups. Chi-squared tests were used to compare identified subgroups with clinical outcomes.

**Results:**

We identified two subgroups of patients in our analysis. Group 1 had a high rate of medical comorbidities causing immunosuppression, requiring vascular access, or both. Group 2 was characterized by a high proportion of people with substance use disorders. Patients in Group 2 were more likely to be readmitted within 6 months than patients in Group 1 (p = 0.03). There was no difference between groups in new paralysis or mortality.

**Discussion:**

While prior studies have examined the SEA patient population as a whole, our research indicates that there are at least two distinct subgroups of patients with SEA. Patients who are younger, with substance use disorder diagnoses, may have longer hospital courses and are at higher risk of readmission within six months. Future research should explore how to best support patients in both groups, and additional implications for subgroup classification on health outcomes, including engagement in care.

## Introduction

Spinal epidural abscesses (SEA) are rare infections with increasing incidence and high morbidity and mortality associated with delayed diagnosis [1–3]. Located in the epidural space, these abscesses can cause nerve compression and damage to vascular structures if untreated. Previous literature has demonstrated that the most common risk factor for SEA is intravenous drug use (IVDU) [4]. However, this has not been associated with increased mortality in patients with SEA [5, 6]. Risk factors for mortality from SEA include diabetes, chronic kidney disease, malignancy, and older age [5–9]. These comorbidities may be a less common presentation of SEA than people with IVDU. As the opioid epidemic continues to affect communities in the United States, physicians are primed to think of a history of IVDU as a primary red flag to prompt a workup for SEA [10]. We sought to identify patient profiles common among patients with SEA, hypothesizing that subgroups would reflect common risk factors for SEA, including IVDU and immunocompromised states.

To identify patient profiles common among patients with SEA, we used latent class analysis. Latent class analysis is a statistical analysis tool which can be used to determine subgroups within a larger data set [11]. Identification of subgroups allows for testing hypotheses between covariates of interest and subgroup assignments. To our knowledge, this analysis type has not been used to study SEA populations. The primary objective was to identify characteristics of subgroups of patients presenting with SEA at a single academic institution in Portland, Oregon. Secondary analyses explored associations between subgroups and new paralysis, in-hospital mortality, and 180-day readmissions.

## Methods

### Study setting and design

Oregon Health & Science University (OHSU) is an academic hospital and Level I Trauma Center in Portland, Oregon. OHSU has 411 adult inpatient beds, and admits over 22,000 adult patients per year [12]. As a tertiary referral center and one of two Level I trauma centers in Oregon, OHSU receives transfers of critically ill patients from across the state, as well as from California, Washington, Idaho, Montana, Wyoming, and Alaska. The severity of SEA may require patients to be transferred to higher levels of care, and SEA patients are likely to be transferred to OHSU.

### Participants

We identified patients from October 2015 to May 2019 with an ICD-10 code of G061. We analyzed data from all patients’ first encounter for a spinal epidural abscess during these years. All patients were at least 18 years old.

### Data collection

We used routinely collected electronic health record data in our analyses. For continuous variables, we collected patient age at hospital encounter and length of stay (if admitted). For binary and categorical variables, we collected sex (male/female), insurance type (Medicaid/Medicare/Self-pay/Other), smoking status (current smoker/non-smoker or past-smoker), and in-hospital death. We report all ICD-10 codes collected for co-morbid conditions in S1 Table. We considered the patient to have new-onset paralysis if an ICD-10 code for plegia was added during the study encounter.

### Data analysis

#### Latent class analysis

We used latent class analysis (LCA) to identify subgroups of patients presenting with spinal epidural abscess. LCA provides two outputs: 1) the proportion of patients belonging to subgroups identified, and 2) the characteristics of subgroups by response probability [13]. We used LCA because LCA uses a top-down approach, allowing the identification of latent classes from the data’s natural distribution, instead of using distance-derived clustering [14]. Additionally, LCA has shown to be superior in identifying clusters versus other clustering techniques, including K-means [15].

We hypothesized that patients would have at least two distinct subgroups by mechanism of spinal epidural abscess development, and tested model fit for one to six subgroups of patients. We consulted with clinical teams and reviewed literature describing demographic factors and diagnoses that were heterogeneous among patients presenting with SEA. Our goal was to identify variables that could explain heterogeneity in patients with SEA. After clinical discussions and literature review, we included the following variables in our latent class analyses: Alcohol Use Disorder (yes/no), Opioid Use Disorder (yes/no), Stimulant Use Disorder (yes/no), Diabetes Mellitus (yes/no), Homeless (yes/no), Active Malignancy (yes/no), Medicaid (yes/no), Age (split into quartiles by distribution pattern).

#### Model selection

We generated 1000 random sets of starting values for our analysis in PROC LCA, and planned to choose the solution with best stability [11, 16] and lowest Bayesian information criterion (BIC), another traditionally used model selection value [17]. We report all model fit statistics in S2 Table.

#### Bivariate analysis

We planned exploratory analyses to compare any assigned classes by demographics and key outcomes (e.g. in-hospital death, paralysis, readmission rates), but did not calculate power for these studies as we were unsure how many clusters our analysis might produce. We planned to use chi-squared tests and Fisher’s exact tests with an alpha of 0.05 to assess differences. We evaluated in-hospital mortality and 180-day readmission rates among patients diagnosed with SEA. We also explored new-onset paralysis because paralysis is particularly debilitating for a medically and socially vulnerable population, and has been examined in a recent study [5].

We used Stata 15 [18] to clean our data, and SAS PROC LCA & LTA [13] and SAS 9.4 [19] to analyze data. SAS and all other SAS Institute Inc. product or service names are registered trademarks or trademarks of SAS Institute Inc., Cary, NC, USA. This project was approved by Oregon Health & Science University Institutional Review Board.

### Ethics statement

This project was approved by Oregon Health & Science University Institutional Review Board (IRB), Study #00018422. The IRB waived the requirement for informed consent as this was routine secondary data analysis.

## Results

**Fig 1:**
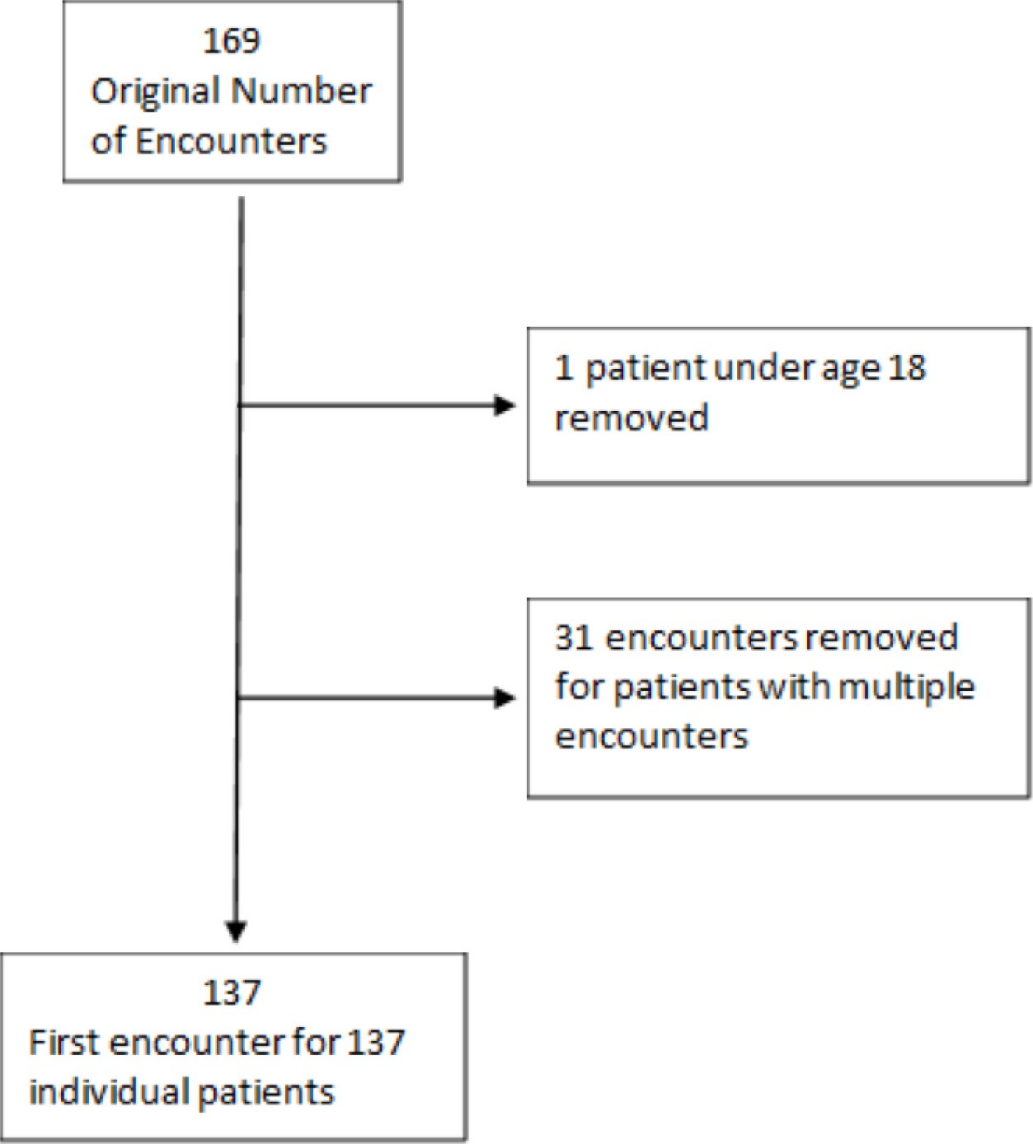
CONSORT diagram of patients diagnosed with a spinal epidural abscess.

Our initial data set included 169 hospital encounters, from which we removed 31 encounters that represented subsequent encounters for patients previously hospitalized with SEA. We assessed data from the first hospitalization of 138 patients. We dropped one patient who was younger than 18 years old; our final cohort included 137 patients (Fig 1).

Using latent class analysis, we identified two subgroups: Group 1 was primarily characterized by the presence of a major medical co-morbidity (n = 70, 52.1%), and Group 2 was primarily characterized by the presence of a substance use disorder (SUD), including IVDU (n = 67, 47.9%) (Table 1).

**Table 1:** Characteristics of participants with spinal epidural abscess, 2015 to 2019*.

	All patients, n = 137	Cluster 1, n = 70	Cluster 2, n = 67	p-values
**Sex** Male	86 (62.8%)	48 (68.6%)	38 (56.7%)	0.151
**Race** White	121 (88.3%)	61 (87.1%)	60 (89.6%)	0.661
**Ethnicity** Hispanic/Latino	4 (2.9%)	2 (2.9%)	2 (3.0%)	0.177
**Direct Admission**	73 (53.3%)	31 (44.3%)	42 (62.7%)	0.031
**Length of Stay**	20.5 (15.6)	14.8 (10.7)	26.5 (17.6)	<0.001
**Current Cigarette Use**	43 (31.4%)	9 (12.9%)	34 (50.7%)	<0.001
**Cocaine Use Disorder**	6 (4.4%)	0 (0%)	6 (9.0%)	0.012
**Sedative Use Disorder**	2 (1.5%)	0 (0%)	2 (3.0%)	0.237
**Cannabis Use Disorder**	2 (1.5%)	0 (0%)	2 (3.0%)	0.237
**HIV Positive**	3 (2.2%)	0 (0%)	3 (4.5%)	0.114
**Chronic Kidney Disease**	13 (9.5%)	13 (18.6%)	0 (0%)	<0.001
**Dialysis Patient**	4 (2.9%)	4 (5.7%)	0 (0%)	0.120
**Cirrhosis**	17 (12.4%)	6 (8.6%)	11 (16.4%)	0.164
**Hepatic Failure**	2 (1.6%)	1 (1.4%)	1 (1.5%)	1.000
**Spinal Defect**	4 (2.9%)	0 (0%)	4 (6.0%)	0.055

*We used posterior probability values to assign patients to a subgroup for the purposes of displaying demographic information in Table 1.

**Fig 2:**
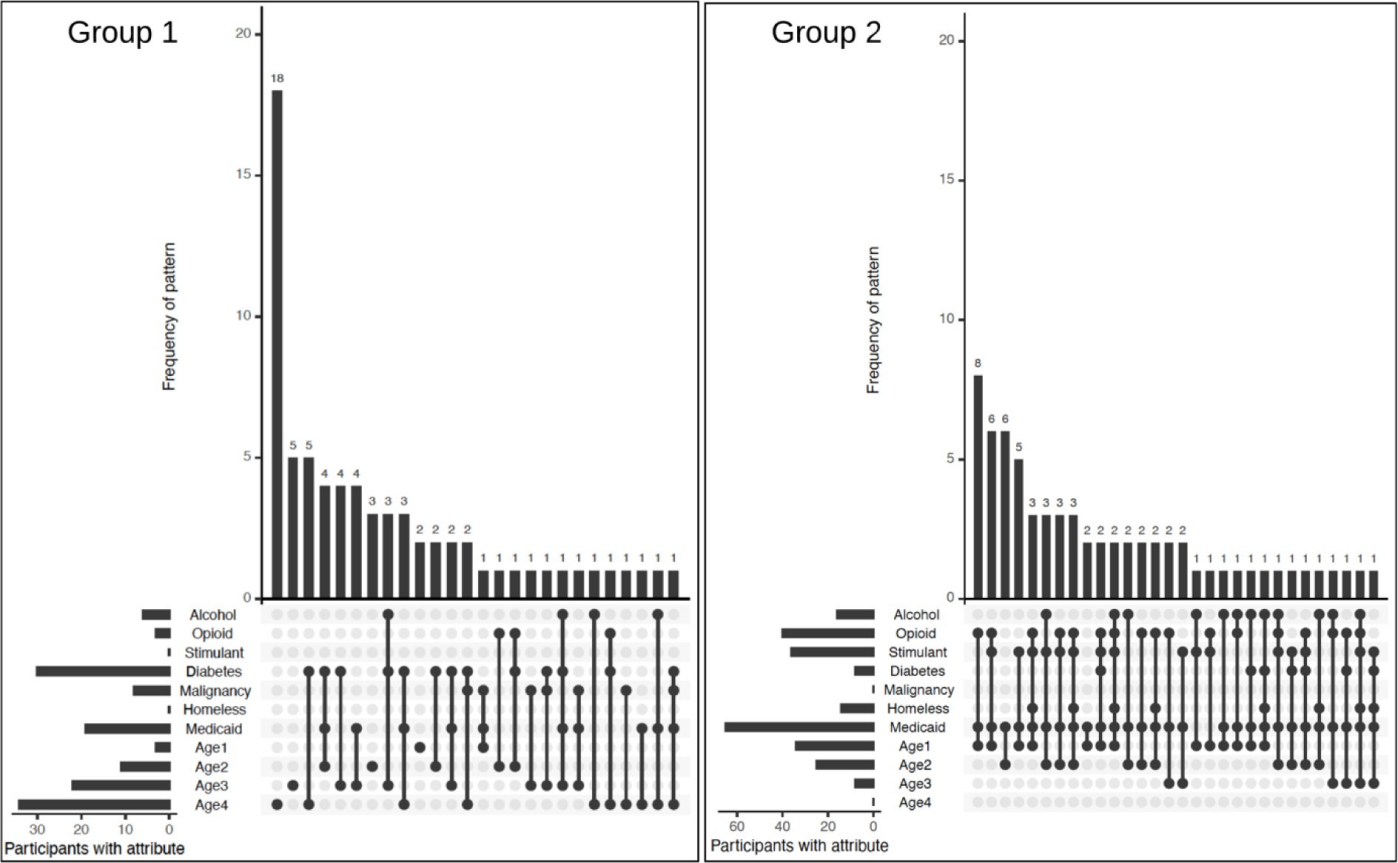
Proportion of patients in each cluster with specific demographic and medical characteristics.

In Group 1, 4.5% of patients had a diagnosis of Opioid Use Disorder, none had a diagnosis of Stimulant Use Disorder, and 9.2% had a diagnosis of Alcohol Use Disorder. Demographically, ages of patients in the medical co-morbidity group were: younger than 48 years (4.6%), between 48 years and 56 years (17.9%), between 57 and 62 years (30.0%), and older than 62 years (47.5%); none were homeless, and 30.2% received Medicaid. 40.9% were diagnosed with Type 2 diabetes mellitus, and 11.0% were diagnosed with active malignancy (Fig 2, Table 2).

**Table 2:** Variables included in latent class analysis of patients with spinal epidural abscess, 2015–2019.

	All patients, n = 137	Cluster 1, n = 70	Cluster 2, n = 67
**Age**	54.1 (14.3)	63.5 (9.7)	44.3 (11.5)
**Homeless**	14 (10.2%)	0 (0%)	14 (20.9%)
**Medicaid Status**	84 (61.3%)	19 (27.1%)	65 (97%)
**Alcohol Use Disorder**	22 (16.1%)	6 (8.6%)	16 (23.9%)
**Opioid Use Disorder**	43 (31.4%)	3 (4.3%)	40 (59.7%)
**Stimulant Use Disorder**	36 (26.3%)	0 (0%)	36 (53.7%)
**Diabetes Mellitus**	38 (27.7%)	30 (42.9%)	8 (11.9%)
**Active Malignancy**	8 (5.8%)	8 (11.4%)	0 (0%)

In Group 2, 60.6% of patients had a diagnosis of Opioid Use Disorder, 54.6% had a diagnosis of Stimulant Use Disorder, and 23.5% had a diagnosis of Alcohol Use Disorder. Demographically, ages of patients in the SUD group were: younger than 48 years (51.3%), between 48 years and 56 years (35.4%), between 57 and 62 years (13.1%), and older than 62 years (0%); 21.3% were homeless, and 95.2% received Medicaid. 13.4% were diagnosed with Type 2 diabetes mellitus, and none were diagnosed with active malignancy (Fig 2, Table 2).

There was a significant difference between the groups for 180-day readmission (Table 3). Five patients (7.1%) in Group 1, versus 13 patients (19.4%) in Group 2, were readmitted within 180-days (p = 0.03). In Group 1, 9 (12.9%) patients developed plegia and 4 (5.7%) patients died in the hospital. In Group 2, 4 (6.0%) patients developed plegia and 2 (3.0%) died in the hospital. Analyses comparing group assignments and the outcomes of new paralysis and in-hospital mortality were not significant.

**Table 3:** Readmission, in-hospital mortality and new plegia by assigned patient groups among patients with SEA, 2015–2019.

	All patients, n = 137	Group 1, n = 70	Group 2, n = 67
**New plegia**	13 (9.5%)	9 (12.9%)	4 (6.0%)
**In-hospital mortality**	6 (4.4%)	4 (5.7%)	2 (3.0%)
**New-onset plegia, in-hospital mortality, or both**	17 (12.4%)	11 (15.7%)	6 (9.0%)
**Readmission within 180 days**	18 (13.1%)	5 (7.1%)	13 (19.4%)

## Discussion

Our analysis shows that the population of patients with SEA comprises two clinically distinct groups. Approximately half of patients with SEA were classified into each group. Group 1 was older than Group 2, with major medical co-morbidities including diabetes and active malignancies. Group 2 had more people with Alcohol Use Disorder, Stimulant Use Disorder, and Opioid Use Disorder, who were also homeless and on Medicaid insurance.

Patients in Group 2 were more likely to be admitted directly through the Emergency Department (ED) rather than transferred to our institution, and had a longer average length of stay (17.6 days versus 10.7 days). All patients with Cocaine Use Disorder, Sedative Use Disorder, Cannabis Use Disorder, and HIV were similarly classified into Group 2, though these variables were not included in latent class analyses. All patients with Chronic Kidney Disease, and those on dialysis, were classified as Group 1 patients. Patients in Group 2 were more likely to be readmitted within 180-days than patients in Group 1 (p = 0.03), but there were no differences in in-hospital mortality or new paralysis among study patients.

We identified patients in Group 2 as having significantly greater 180-day readmission than patients in Group 1. Prior work finds that hepatic disease (due to a history of alcohol abuse or viral hepatitis) and immunocompromise are both significantly and independently associated with an increased 90-day readmission rate [20]. Our own work identified both hepatic disease and immunocompromised status as components of profiles for SEA. The most common reason for SEA readmission in the literature is infection, and a higher rate of severe infection in a younger, more immunocompetent population demands explanation [20]. We suspect that patients with SUDs may be readmitted at a higher rate due to a number of social factors, including homelessness, stigma, and reduced outpatient follow-up [21]. We urge particular attention to these factors in discharge planning for this population.

We expected there to be an association between Group 1 classification and mortality, as Group 1 demographics (older age, active malignancy, diabetes, and chronic kidney disease) have been associated with in-hospital, 30-day, and 90-day mortality [5–9]. Diabetes and malignancy are also associated with failure of medical therapy in these patients [22]. However, in our analysis, membership in Group 1 did not predict death, although Group 1 had a non-significantly higher mortality rate. This is a surprising finding, as Group 1 patients were older and had more comorbid conditions, but may be because of limited power in this small sample to detect a difference.

Group 1 patients also had a non-significantly greater risk of developing new paralysis. We suspect that in this less immunocompetent population, the risk of a large or rapidly expanding abscess is greater. These patients may also be at risk of delayed identification because they lack the most visible risk factor for SEA: IVDU.

Our study has several limitations. First, the source of our data, billing records, excludes clinically important data. In particular, initial lab values are a crucial prognostic indicator for patients with SEA. However, this research has been well-documented in the literature, and our primary goal in this research was to identify subgroups of patients with SEA, and seek clarification if these subgroups were related to key clinical outcomes. Second, risk factors were identified using ICD-10 codes, which likely underestimate patient conditions. Third, our sample size is limited by the relative rarity of SEA and the use of a single academic medical center to evaluate data. Fourth, a prior calculation of sample size required to show a meaningful relationship between outcomes for identified classes was not performed because the number of classes and expected effect size was not known, and we may have been underpowered to show a real difference in new paralysis and mortality. Fifth, clustering, like any analysis, can produce different results in different datasets. Future research should validate these clusters in national datasets with additional SEA patients. Sixth, because of the small sample size, we did not split our dataset into train and test sets to validate our models. Additionally, PROC LCA Bootstrap (in SAS) does not allow inclusion of non-binary variables, and is one of only two programs that includes an option for bootstrapping after LCA at all [23, 24]. Thus, we were unable to use bootstrapping to validate our models. Larger datasets must build on this work to use robust validation methods to evaluate model fit. Finally, our institution, located in Portland, Oregon, has limited racial and ethnic diversity, which may make generalizability of our findings to the broader population challenging.

There are important implications from this research. First, the two groups identified in this analysis reflect two distinct patient populations with SEA. While IVDU is likely the most recognizable risk factor for SEA among ED physicians, approximately half of our patients were classified into a group marked by older age and diagnoses of diabetes and active malignancy. Second, readmission rate was higher among patients with SEA. Future research with SEA patients with IVDU is needed to identify how to best support these patients during first and subsequent readmissions, and to better understand why these patients are at higher risk of readmission. Finally, there may be clinically important differences in microbiological etiology of SEA between these two groups, including differences in the incidence of *Pseudomonas* infections, which could inform empiric antibiotic choice [1, 25].

Patients with and without IVDU have not been shown to differ significantly in mortality or paralysis outcomes, in our study or in prior research [5, 6]. However, separate consideration of our identified groups will facilitate better outcome predictions, risk factor identification, and treatment needs for these distinct patient populations. Patients with SUDs presenting with SEA may benefit from the involvement of additional inpatient support for SUD, as can be offered through addiction consult services [26]. Such services can help reduce substance use [27] and improve outpatient engagement with SUD treatment [28], and may help improve patient trust and engagement in care generally.

## Supporting information

S1 Table ICD-codes used to identify comorbid conditions. (DOCX)

S2 Table Model fits for tested latent class analyses by number of clusters. (DOCX)

## References

[1] Darouiche R.O., Spinal epidural abscess. N Engl J Med, 2006 355(19): p. 2012–20. 17093252 10.1056/NEJMra055111

[2] Vakili M. and Crum-Cianflone N.F., Spinal Epidural Abscess: A Series of 101 Cases. Am J Med, 2017 130(12): p. 1458–1463. 28797646 10.1016/j.amjmed.2017.07.017

[3] Davis D.P., et al, The clinical presentation and impact of diagnostic delays on emergency department patients with spinal epidural abscess. J Emerg Med, 2004 26(3): p. 285–91. 15028325 10.1016/j.jemermed.2003.11.013

[4] Turner A., et al, Management of cervical spine epidural abscess: a systematic review. Ther Adv Infect Dis, 2019 6: p. 2049936119863940. 31367375 10.1177/2049936119863940 PMC6643182

[5] Shah A.A., et al, Development of Predictive Algorithms for Pre-Treatment Motor Deficit and 90-Day Mortality in Spinal Epidural Abscess. J Bone Joint Surg Am, 2018 100(12): p. 1030–1038. 29916930 10.2106/JBJS.17.00630

[6] Zafonte R.D., et al, Spinal epidural abscess: study of early outcome. J Spinal Cord Med, 2003 26(4): p. 345–51. 14992335 10.1080/10790268.2003.11753704

[7] Du J.Y., et al, 30-day Mortality Following Surgery for Spinal Epidural Abscess: Incidence, Risk Factors, Predictive Algorithm, and Associated Complications. Spine (Phila Pa 1976), 2019 44(8): p. E500–E509. 30234819 10.1097/BRS.0000000000002875

[8] Karhade A.V., et al, Development of machine learning algorithms for prediction of mortality in spinal epidural abscess. Spine J, 2019 19(12): p. 1950–1959. 31255788 10.1016/j.spinee.2019.06.024

[9] Schoenfeld A.J. and Wahlquist T.C., Mortality, complication risk, and total charges after the treatment of epidural abscess. Spine J, 2015 15(2): p. 249–55. 25241303 10.1016/j.spinee.2014.09.003

[10] DiGiorgio A.M., et al, The increasing frequency of intravenous drug abuse-associated spinal epidural abscesses: a case series. Neurosurg Focus, 2019 46(1): p. E4. 10.3171/2018.10.FOCUS18449 30611170

[11] Lanza L.C.xa.S., *Latent Class and Latent Transition Analysis: With Applications in the Social, Behavioral, and Health Sciences*. 2010, Hoboken, NJ: John Wiley & Sons, Inc.

[12] OHSU, OHSU Facts | OHSU. 2019.

[13] Lanza S, D.J., Huang L, Wagner A, Collins LM, *PROC LCA & PROC LTA Users' Guide Version 1*.*3*.*2*. The Methodology Center, Penn State University, 2015.

[14] *Latent Class Analysis vs. Cluster Analysis—differences in inferences?* 2017 [cited 2020; Available from: https://stats.stackexchange.com/questions/122213/latent-class-analysis-vs-cluster-analysis-differences-in-inferences.

[15] Vermunt J. and Magidson J., *Latent Class Cluster Analyses*. 2002.

[16] Lanza S.T. and Bray B.C., Transitions in Drug Use among High-Risk Women: An Application of Latent Class and Latent Transition Analysis. Adv Appl Stat Sci, 2010 3(2): p. 203–235. 21921977 PMC3171700

[17] Nylund K.L., A.T., & Muthen B.O., Deciding on the Number of Classes in Latent Class Analysis and Growth Mixture Modeling: A Monte Carlo Simulation Study. Structural Equation Modeling: A Multidisciplinary Journal, 2007 14(4): p. 535–569.

[18] StataCorp, *Stata Statistical Software: Release 15*. College Station, TX: StataCorp LLC 2017.

[19] SAS, SAS 9.4. 2019.

[20] Longo M., et al, Readmission after spinal epidural abscess management in urban populations: a bi-institutional study. J Neurosurg Spine, 2019: p. 1–8. 10.3171/2019.8.SPINE19790 31756697

[21] Ferari C.S., et al, Implications of Drug Use Disorders on Spine Surgery. World Neurosurg, 2020. 10.1016/j.wneu.2019.12.177 PMC7556334 31926361

[22] Shah A.A., et al, Nonoperative Management of Spinal Epidural Abscess: Development of a Predictive Algorithm for Failure. J Bone Joint Surg Am, 2018 100(7): p. 546–555. 29613923 10.2106/JBJS.17.00629

[23] Dziak J.J. and Lanza S.T., *LcaBootstrap SAS macro users’ guide (version 4*.*0)*. 2016, The Methodology Center, Penn State: University Park p. 3.

[24] Dziak J.J., Lanza S.T., and Tan X., Effect Size, Statistical Power and Sample Size Requirements for the Bootstrap Likelihood Ratio Test in Latent Class Analysis. Struct Equ Modeling, 2014 21(4): p. 534–552. 25328371 10.1080/10705511.2014.919819 PMC4196274

[25] Lorson W.C., Heidel R.E., and Shorman M.A., Microbial Epidemiology of Infectious Endocarditis in the Intravenous Drug Abuse Population: A Retrospective Study. Infect Dis Ther, 2019 8(1): p. 113–118. 30673991 10.1007/s40121-019-0232-7 PMC6374230

[26] Priest K.C. and McCarty D., Role of the Hospital in the 21st Century Opioid Overdose Epidemic: The Addiction Medicine Consult Service. J Addict Med, 2019 13(2): p. 104–112. 30608266 10.1097/ADM.0000000000000496 PMC6417955

[27] Wakeman S.E., et al, Inpatient Addiction Consultation for Hospitalized Patients Increases Post-Discharge Abstinence and Reduces Addiction Severity. J Gen Intern Med, 2017 32(8): p. 909–916. 28526932 10.1007/s11606-017-4077-z PMC5515798

[28] Englander H., et al, Inpatient Addiction Medicine Consultation and Post-Hospital Substance Use Disorder Treatment Engagement: a Propensity-Matched Analysis. J Gen Intern Med, 2019. 10.1007/s11606-019-05251-9 PMC6854181 31410816

