# Supplementary material for: Evaluating new paralysis, mortality, and readmission among subgroups of patients with spinal epidural abscess: A latent class analysis
Source: PLoS One. 2020 Sep 11;15(9):e0238853. doi: 10.1371/journal.pone.0238853 (PMC7485888; doi:10.1371/journal.pone.0238853)
Supplement: S1 Table — (DOCX) [file pone.0238853.s001.docx]

**S1 Table. ICD-codes used to identify comorbid conditions.**

| Condition | ICD-10 Code(s) |
| --- | --- |
| Plegia | G8101, G8194, G8220, G8221, G8250, G8311 |
| Homeless | Z59.0 |
| Current Cigarette Use | F17210 |
| Alcohol Use Disorder | F10.x (excluding F1021) |
| Opioid Use Disorder | F11.x |
| Stimulant Use Disorder | F15.x |
| Cocaine Use Disorder | F14.x |
| Sedative Use Disorder | F13.x |
| Hallucinogen Use Disorder | F16.x |
| Inhalant Use Disorder | F18.x |
| Cannabis Use Disorder | F12010 |
| HIV Positive | B20, Z21 |
| Active Malignancy | C7a019 |
| Diabetes Mellitus | E11 |
| Chronic Kidney Disease | I12.x, I13.x, N18.x |
| Dialysis Patient | Z99.2 |
| Cirrhosis | K70.3, K70.31, K74.6, K74.69 |
| Hepatic Failure | K70.4x |
| Spinal Defect | Q0700, Q675, Q7649 |

*Stimulant Use Disorder likely reflects methamphetamine use, due to the widespread use of methamphetamine on the West Coast, and the distinct diagnosis of Cocaine Use Disorder.
