# Supplementary material for: Evaluating new paralysis, mortality, and readmission among subgroups of patients with spinal epidural abscess: A latent class analysis
Source: PLoS One. 2020 Sep 11;15(9):e0238853. doi: 10.1371/journal.pone.0238853 (PMC7485888; doi:10.1371/journal.pone.0238853)
Supplement: S2 Table — (DOCX) [file pone.0238853.s002.docx]

**S2 Table. Model fits for tested latent class analyses by number of clusters.**

|  | Log-likelihood | G-squared | AIC | BIC | CAIC | Adjusted BIC | Entropy | Degrees of freedom | Solution Stability |
| --- | --- | --- | --- | --- | --- | --- | --- | --- | --- |
| 1-Cluster | -661.99 | 317.21 | 337.21 | 366.41 | 376.41 | 334.77 | 1.00 | 501 | 100.0% |
| 2-Cluster | -574.39 | 142.00 | 184.00 | 245.32 | 266.32 | 178.88 | 0.82 | 490 | 100.0% |
| 3-Cluster | -568.05 | 129.31 | 193.31 | 286.75 | 318.75 | 185.52 | 0.73 | 479 | 51.20% |
| 4-Cluster | -561.21 | 115.64 | 201.64 | 327.20 | 370.20 | 191.17 | 0.78 | 468 | 11.40% |
| 5-Cluster | -555.27 | 103.76 | 211.76 | 369.44 | 423.44 | 198.60 | 0.81 | 457 | 13.90% |
| 6-Cluster | -547.54 | 88.30 | 218.30 | 408.10 | 473.10 | 202.46 | 0.85 | 446 | 5.20% |
